# Management of symptomatic irreversible pulpitis using irrigation agitation techniques in anterior teeth

**DOI:** 10.6026/973206300191129

**Published:** 2023-12-31

**Authors:** Ramdhas Annapurani, Pasupathy Shakunthala, Selvaraj Sharmila, Subramanian Dhanalakshmi, Jayavel Nandhakumar, Manoharan Kaladevi

**Affiliations:** 1 Independent Restorative Dentist and Endodontist, Chennai, Tamilnadu, India; 2 Department of Conservative Dentistry and Endodontics, Tamil Nadu Government Dental College and Hospital, Chennai, Tamilnadu, India; 3 Government Nagapattinam Medical College, Tamilnadu, India

**Keywords:** Conventional irrigation, post-operative pain, endovac irrigation, negative apical pressure

## Abstract

Endodontic pain, a common complication after root canal treatment, affects 2.5% to 60% of patients. Therefore, it is of interest to compare apical negative pressure irrigation (EndoVac) with conventional needle irrigation to assess their impact on postoperative pain in permanent anterior teeth with symptomatic irreversible pulpitis. Fifty patients were randomly assigned to either the EndoVac or needle irrigation group. Pre and post-operative pain levels were assessed using a Visual Analog Scale and the amount of Ibuprofen taken was recorded. At 12-, 24-, and 48-hour intervals, the EndoVac group reported significantly less pain than the needle irrigation group. The needle irrigation group also required more Ibuprofen. The apical negative pressure irrigation system (EndoVac) resulted in significantly less postoperative pain and reduced the need for analgesic medication than the conventional needle irrigation protocol.

## Background:

Many patients visit the dentist due to varying degrees of pain, which is most likely to be of an endodontic origin. Endodontic treatment involves managing all pre-, intra-, and postoperative symptoms. Postoperative endodontic pain, a therapeutic complication that can become chronic, is of significant concern to patients and dentists [1 2-3]. Postoperative pain is extremely prevalent, affecting anywhere between 2.5% to over 60% of patients who have had endodontic procedures and it tends to get worse between 6 and 12 hours after treatment, peaking at around 40% in 24 hours before dropping to 11% one week later. Pain after endodontic treatment has multifactorial etiological factors, including mechanical, chemical or microbiological damage to the pulp or periradicular tissues. Infected root canals are among the most frequent causes of endodontic discomfort [4- 5]. Complete chemo-mechanical debridement of canals is reportedly quite difficult despite the use of contemporary rotary instruments. Many studies employing micro computed tomography scanning have demonstrated that the majority of the areas in the main root canal walls are unaffected by the instruments. These regions may contain tissue fragments, multi-species microbial biofilms and microbial by-products, which could lead to reinfection or continuous periradicular inflammation. Irrigation is essential to root canal therapy to eliminate contaminated necrotic tissue before and during the procedure. Despite exploring numerous irrigation solutions and techniques, no guaranteed approach is available for successful root canal treatment. This emphasizes the need to continually explore novel methods for suitably cleaning and disinfecting the root canal. Therefore, it is of interest to compare the apical negative pressure irrigation technique (EndoVac) with the conventional needle technique to assess their impact on postoperative pain following root canal treatment in permanent anterior teeth [5- 6,7,8].

## Materials and Methods:

The prospective randomized clinical trial was conducted in the Department of Conservative Dentistry and Endodontics, Tamil Nadu Government Dental College and Hospital, Chennai, India. This clinical trial was approved by the Institutional Review Board (IRB) for ethical clearance (IRB Ref No. 02/IERB/2020-R 01/10/2020). Fifty adult patients who met the inclusion and exclusion criteria were selected as cohorts for this study.

## Inclusion criteria:

Patients aged 18-50 years with no history of systemic disease, with periodontal healthy oral tissues, and a clinical diagnosis of symptomatic irreversible pulpitis in anterior teeth was included.

## Exclusion criteria:

Patients with analgesics, anti-inflammatory medicines, steroids, opioids, or NSAIDs within 12 hours before treatment, negative pulp vitality tests or pain upon percussion, teeth with root curvature greater than 25 degrees, calcified root canals, open apices, periapical radiolucency, root resorption, para-functional habits, weakened periodontium, or significant malocclusions linked to traumatic occlusion, patients who were nursing, pregnant, medically compromised or allergic to NSAIDs or local anesthetics were excluded. Clinical examinations and radiographs were performed on all patients. Symptomatic irreversible pulpitis was diagnosed based on clinical findings. Patients were informed about the treatment options and agreed to participate. Oral prophylaxis was performed before treatment began. Pre- and postoperative pain was assessed using a VAS questionnaire. Patients were randomly assigned into either group: Group 1: (n=25) Final irrigation agitation using conventional needle irrigation technique (control). Group 2: (n=25) Final irrigation agitation using the true apical negative pressure irrigation (EndoVac system, Discus Dental, Culver City, CA, USA).

## Root canal procedure:

Root canal treatment was performed on both groups by the same operator. Canals were prepared, instrumented and enlarged with intermittent irrigation. The final irrigation protocol varied between groups, with the operator unaware of the irrigant agitation system until final irrigation to minimize bias. Patients received ibuprofen prescriptions and were instructed to contact the researcher for pain management.

## Postoperative pain assessment:

Postoperative pain was assessed via telephone at 6, 12, 24, and 48 hours after endodontic treatment. Participants were trained to use a questionnaire and Visual Analog Scale (VAS) to mark discomfort. Pain levels were classified as no pain, mild, moderate or severe and the total number of analgesic pills taken during the follow-up period was also recorded.

## Statistical analysis:

Statistical analysis was performed using IBM SPSS 26.0 software. Age, sex and analgesic intake were analyzed using the Chi-square test. The Mann-Whitney test was used to analyse the statistical significance between the two groups at various time points. The Shapiro-Wilks test and Kolmogorov-Smirnov test (Lilliefors Significance Correction) were used to test the data for normality. A p-value < 0.05 was considered statistically significant for all tests.

## Results:

Table 1 gives a descriptive review of the cohorts' demographics and pain analysis. The mean age group of the patients included in the study was 36.12± 6.67 in the Conventional needle group and 36.04±10.46 in the EndoVac group. There is no significant difference in the age group of patients. Of the participants, 74% (n=37) were females and 26% (n=13) were males. The participants of both genders were evenly distributed within the groups. According to the Pearson Chi-square test, there was no significant difference in their distribution among the groups (p=0.747).

## Pre-operative VAS pain analysis:

All the patients included had moderate levels (VAS 4-6) of pain before the start of the procedure. The two groups have no significant difference in the pre-operative pain scores (p>0.05).

## Postoperative pain analysis:

The analysis after irrigation showed that in the EndoVac group, the maximum pain score experienced was 4, whereas, in the Conventional needle group, the level reached 9. The patients in the EndoVac group had reported no pain after 24 hours postoperatively, while in the conventional needle irrigation group, the mean score reported at 24 hours was 1.36 and at 48 hours was 0.48.

## Inferential statistics for baseline characteristics & comparison of pain intensity between two groups at different time points:

The intergroup comparison showed an improved reduction in pain in the EndoVac group compared to the conventional needle group at 6, 12, 24, and 48 hours (Table 2). But the difference is insignificant at 6 hours (p>0.05). However, the difference in the occurrence of pain is statistically significant amongst the two groups at 12, 24, and 48 hours of the postoperative period and the points assessed (p<0.05). At 24 hours, the groups had an extremely significant difference in reported pain (p<0.001). No significant difference exists between the patient's age group and pre-operative pain scores (p>0.05).

**Figure 1:**
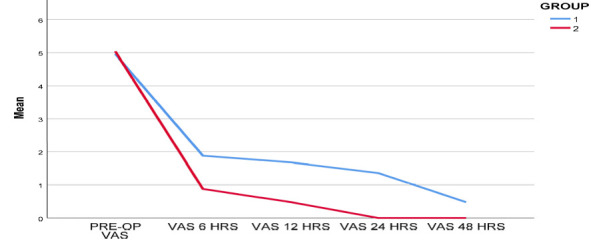
: Postoperative pain over the time duration for both treatment groups

Figure 1 plots the postoperative pain over the time duration for both treatment groups. In both groups, there is a steep reduction in the mean postoperative pain observed in the first 6 hours, followed by a gradual decline in the next follow-up periods, 12, 24 and 48 hours. In the EndoVac group, the mean value reported reached almost 0 at 12 hours, and no pain was observed after 24 hours. There is an observable difference in pain reduction between the groups from 6 to 48 hours.

## Intragroup analysis:

**Figure 2:**
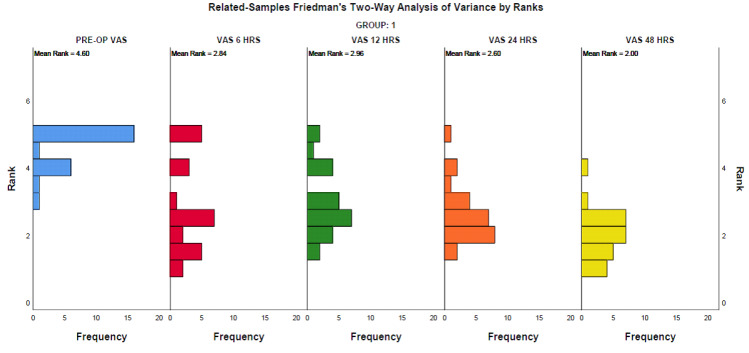
Group 1 VAS at different intervals

**Figure 3:**
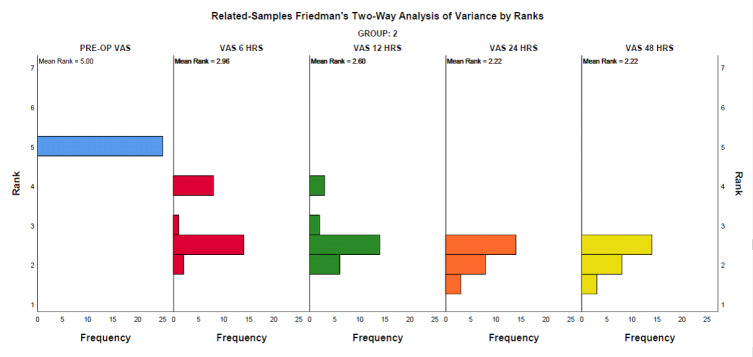
: Group 2 VAS at different intervals

Pre-op VAS was compared with postoperative VAS at 6, 12, 24 and 48 hours. The ordinal data obtained were subjected to related samples Friedman's Two-Way Analysis of Variance by Ranks (with Bonferroni correction) for intragroup analysis to assess significant change in study parameters over different time points assessed. In both groups, i.e., the Conventional needle group and the EndoVac group, a significant difference was observed in comparing pre-operative pain values with 6, 12, 24 and 48 hours (p<0.01). This shows a significant reduction in pain observed 6 hours after the endodontic procedure in both groups. Figure 2 and Figure 3 depict the frequency distribution in median pain scores in Group 1 and Group 2 respectively.

## Analgesic medication intake:

Of the total participants of the study, only 20% (n=10) had taken analgesics. Among the ten patients who took analgesics, eight belonged to the Conventional needle irrigation group, and only two belonged to the EndoVac group. Pearson Chi-square test analysis showed a significant difference in the analgesics taken between the two groups (p=0.034).

## Discussion:

Endodontic infections are caused by bacterial invasion of the dental pulp, causing inflammation and infection. Root canal therapy (RCT) is crucial for managing these infections, involving instrumentation and irrigation to remove infected tissue and clean the root canal system. Irrigation is essential for the success of RCT and can be increased using manual dynamic agitation, ultrasonic activation or laser activation. The EndoVac, a negative-pressure irrigation system introduced by John Schoeffel, creates negative pressure, drawing irrigation fluid apically. Postoperative pain can be multifactorial, but factors such as patient age, tooth type, root canal anatomy, pulpal status, pre-operative pain level and working length maintenance are considered [9- 10,11].

In the current clinical trial, regardless of the final irrigation agitation system employed the postoperative pain score values were significantly lower than pre-operative, and various investigators have noted this conclusion [12, 13]. Studies comparing the conventional needle irrigation approach to other irrigation methods have revealed that patients who had conventional irrigation experienced more postoperative pain [13- 14,15].

In this clinical trial, postoperative pain intensity was significantly higher among patients in the conventional needle irrigation group than those in the EndoVac group at 12-, 24-, and 48-hour intervals (p<0.05). In light of the discoveries of this investigation, the null hypothesis was rejected. Patients experienced less pain on irrigation using the EndoVac irrigation system than conventional needle irrigation.

Research showed that apical negative-pressure irrigation has the potential to achieve better microbial control than conventional irrigation delivery systems [16]. A study performed a comparative evaluative study on 30 permanent maxillary central incisors for the efficacy of an Endovac irrigation system with conventional needle irrigation in removing the smear layer from the root canal. The study noted a significantly better removal of the smear layer from the apical third of the root canal using Endovac than Conventional needle irrigation [17]. Another study found significantly better debridement at 1 mm from working length using the EndoVac than needle irrigation [18]. Our study noted that EndoVac was significantly efficient in postoperative pain reduction, which could be attributed to better canal debridement, as noted by the above studies.

In the literature review, Nivedhitha and Swarna analyzed the randomized clinical trials employing different irrigant activation techniques and their efficacy in postoperative pain management. Their review noted that, according to all clinical investigations, irrigation activation using sonic, ultrasonic, laser and manual dynamic agitation decreased postoperative pain. Regarding postoperative pain, the negative pressure irrigation device EndoVac outperforms conventional needle irrigation [14].

One study compared the level of postoperative pain after root canal therapy using endodontic needle irrigation with the negative apical pressure device EndoVac. At all-time intervals evaluated during the study, pain experience with the EndoVac was significantly lower than when using the needle irrigation. Within 24 hours of postoperative analysis, the intake of analgesics was significantly lower in the EndoVac group. In agreement with the present study results, Gondim Jr et al. also found that EndoVac patients experienced lesser postoperative pain [19].

The findings of the current study concur with Tpocuglu's randomized clinical trial. Researchers in Turkey tested the EndoVac system and conventional needle irrigation for their effects on postoperative pain in mandibular molar teeth with symptomatic irreversible pulpitis. EndoVac system outperformed conventional irrigation in reducing postoperative pain among one hundred sixteen patients with symptomatic irreversible pulpitis [15].A study which examined EndoVac, needle irrigation and passive ultrasonic irrigation on postoperative pain, also produced results similar to the findings of the present study. Postoperative pain values were lowest in the EndoVac group [20].

The current study demonstrated that the EndoVac irrigation device effectively alleviates postoperative discomfort in adult patients with symptomatic irreversible pulpitis who underwent root canal therapy. As a result, we also observed a reduced analgesic intake to mitigate postoperative pain in the EndoVac treatment cohorts. Given that this clinical trial was the first in-vivo study to compare the impact of fluid pressure kinematics on postoperative pain using EndoVac and conventional needle irrigation in permanent maxillary anterior teeth, additional in-vivo studies contrasting the apical extrusion and pain in single-rooted and curved natural teeth may be required to support the findings of our current study.

## Conclusion:

For endodontic treatment to be successful, bacteria must be removed during cleaning and shaping. The irrigant must be used effectively to work in the root canals to the fullest extent possible. Physically sanitizing or cleaning every component of a root canal system is impossible. Irrigation is the only method that can adequately clean these areas. The final irrigation regimen is crucial and needs further research to enhance the success of root canal therapy. The EndoVac irrigation system resulted in significantly less postoperative pain than the conventional needle irrigation method. Patients in the EndoVac group also had significantly lower analgesic usage than patients in the conventional needle irrigation group.

## Figures and Tables

**Table 1:** Patient's age distribution and pain levels in both groups

**Method**	**Variables**	**Mean**	**SD**	**Median**	**Minimum**	**Maximum**
Group 1	Age	36.12	6.673	35	24	49
	Pre-op VAS	4.96	0.735	5	4	6
	VAS 6 hours	1.88	3.073	0	0	9
	VAS 12 hours	1.68	2.076	0	0	7
	VAS 24 hours	1.36	2.018	1	0	8
	VAS 48 hours	0.48	1.418	0	0	7
Group 2	Age	36.04	10.462	40	18	50
	Pre-op VAS	5.04	0.676	5	4	6
	VAS 6 hours	0.88	1.301	0	0	4
	VAS 12 hours	0.48	1.085	0	0	4
	VAS 24 hours	0	0	0	0	0
	VAS 48 hours	0	0	0	0	0

**Table 2:** The statistics for inter-group comparison

	**Age**	**VAS**				
		**Pre-op VAS**	**6 hours**	**12 hours**	**24 hours**	**48 hours**
Mann-Whitney U	273	293.5	287	212.5	137.5	237.5
Wilcoxon W	598	618.5	612	537.5	462.5	562.5
Z	-0.767	-0.404	-0.568	-2.302	-4.298	-2.582
Asymp. Sig (2-tailed)	0.443	0.686	0.57	0.21	0	0.1
